# Identification and analysis of a glutamatergic local interneuron lineage in the adult *Drosophila* olfactory system

**DOI:** 10.1186/2042-1001-1-4

**Published:** 2011-01-26

**Authors:** Abhijit Das, Albert Chiang, Sejal Davla, Rashi Priya, Heinrich Reichert, K VijayRaghavan, Veronica Rodrigues

**Affiliations:** 1 Department of Biological Sciences, Tata Institute of Fundamental Research, Homi Bhabha Road, Mumbai-400005, India; 2 National Centre for Biological Sciences, TIFR, UAS-GKVK Campus, Bangalore-560065, India; 3 Biozentrum, University of Basel, Basel, Switzerland

## Abstract

**Background:**

The antennal lobe of *Drosophila* is perhaps one of the best understood neural circuits, because of its well-described anatomical and functional organization and ease of genetic manipulation. Olfactory lobe interneurons - key elements of information processing in this network - are thought to be generated by three identified central brain neuroblasts, all of which generate projection neurons. One of these neuroblasts, located lateral to the antennal lobe, also gives rise to a population of local interneurons, which can either be inhibitory (GABAergic) or excitatory (cholinergic). Recent studies of local interneuron number and diversity suggest that additional populations of this class of neurons exist in the antennal lobe. This implies that other, as yet unidentified, neuroblast lineages may contribute a substantial number of local interneurons to the olfactory circuitry of the antennal lobe.

**Results:**

We identified and characterized a novel glutamatergic local interneuron lineage in the *Drosophila* antennal lobe. We used MARCM (mosaic analysis with a repressible cell marker) and dual-MARCM clonal analysis techniques to identify this novel lineage unambiguously, and to characterize interneurons contained in the lineage in terms of structure, neurotransmitter identity, and development. We demonstrated the glutamatergic nature of these interneurons by immunohistochemistry and use of an enhancer-trap strain, which reports the expression of the *Drosophila* vesicular glutamate transporter (DVGLUT). We also analyzed the neuroanatomical features of these local interneurons at single-cell resolution, and documented the marked diversity in their antennal lobe glomerular innervation patterns. Finally, we tracked the development of these dLim-1 and Cut positive interneurons during larval and pupal stages.

**Conclusions:**

We have identified a novel neuroblast lineage that generates neurons in the antennal lobe of *Drosophila*. This lineage is remarkably homogeneous in three respects. All of the progeny are local interneurons, which are uniform in their glutamatergic neurotransmitter identity, and form oligoglomerular or multiglomerular innervations within the antennal lobe. The identification of this novel lineage and the elucidation of the innervation patterns of its local interneurons (at single cell resolution) provides a comprehensive cellular framework for emerging studies on the formation and function of potentially excitatory local interactions in the circuitry of the *Drosophila* antennal lobe.

## Background

A relatively small number of genetic and structural modules (neural lineages) make up the *Drosophila* central nervous system [1], with the central brain formed by approximately 100 bilaterally symmetric lineages [2]. Each lineage arises from an asymmetrically dividing neural stem cell, called a neuroblast, which is born during embryonic life [3]. Three of these central brain neuroblasts, denoted as anterodorsal, lateral and ventral, are thought to give rise to most of the interneurons of the adult antennal lobe [4-6]. Each of these neuroblasts initially generates a small set of primary neurons during embryogenesis, which form the larval antennal lobe, and subsequently during larval development produces a larger set of lineage-related secondary (adult-specific) neurons that differentiate during metamorphosis to form the mature olfactory circuitry of the adult [7-9].

The *Drosophila* adult antennal lobe consists of dense glomeruli that are innervated by glomerulus-specific axons of the olfactory sensory neurons (OSNs), and by local interneurons (LNs) and projection neurons (PNs) [10]. Whereas the anterodorsal and ventral neuroblast lineages comprise PNs [4], the lateral neuroblast lineage consists of a complex and heterogenous set of uniglomerular PNs and multiglomerular atypical PNs, and a diverse range of LNs that are mostly GABAergic, with a smaller number of cholinergic types (and LNs with uncharacterized neurotransmitter identity) [5,11,12]. Although many of the known central olfactory neurons are generated from these three identified neuroblast lineages, both functional and structural considerations indicate that other hitherto unidentified neuroblast lineage(s) that contribute interneurons to the olfactory system in the antennal lobe are likely to exist.

Neurophysiological studies on olfactory information processing have identified a dense network of functionally excitatory lateral connections in the antennal lobe that distributes odour-evoked excitation between channels in the glomeruli [13,14]. Indeed, this local lateral excitatory input to neurons in the antennal lobe can be sufficiently strong to trigger action potentials in the olfactory PNs. LNs of the lateral neuroblast lineage are unlikely to mediate all of these pervasive excitatory interactions, because most of them are inhibitory and have a GABAergic neurotransmitter phenotype, whereas only a small subset of these LNs are cholinergic [12,15]. Moreover, in a recent neuroanatomical analysis of LN diversity in the antennal lobe, several novel LN types were identified, and some of these had their somata in a ventrally located cluster, suggesting that they are not derived from the lateral neuroblast [11]. A subset of these LNs is apparently glutamatergic, and their lineage of origin is currently unknown.

Given the role of glutamatergic signalling in olfactory information processing in the antennal lobe during development and adult function [16-18], and in view of the current lack of information on the complete set of neuroblast lineages that generate the antennal lobe circuitry, we set out to determine the neuroblast lineage of origin of the glutamatergic LNs. We identified a novel neuroblast that generates these olfactory LNs, and we provide evidence that this lineage consists exclusively of local glutamatergic interneurons that innervate the antennal lobe.

In this analysis we used the 'enhancer-trap' line Gal4-*OK371*[19] together with immunocytochemistry to visualize a discrete cluster of putative glutamatergic LNs located between the clusters of neurons that comprise the lateral and ventral neuroblast lineages. Using the dual expression control MARCM (mosaic analysis with a repressible cell marker) technique [20] and pharmacological (hydroxyurea) neuroblast ablation, we then found that the lineage of these LNs is distinct from that of the ventral lineage PNs and lateral lineage PNs and LNs [4-6,12]. Subsequently, we analyzed the developmental origin of these LNs and characterized their neuroanatomical properties using single-cell MARCM labelling techniques [21]. Taken together, our experiments identified and characterized a novel fourth neuroblast lineage that contributes glutamatergic LNs to the adult olfactory system of *Drosophila*. We posit that many of the excitatory circuit elements involved in central coding and processing of olfactory information in the glomerular antennal lobe system [13,14] may derive from this lineage.

## Results

### Gal4-*OK371* labelling identifies a glutamatergic cell cluster in the antennal lobe

**Figure 1:**
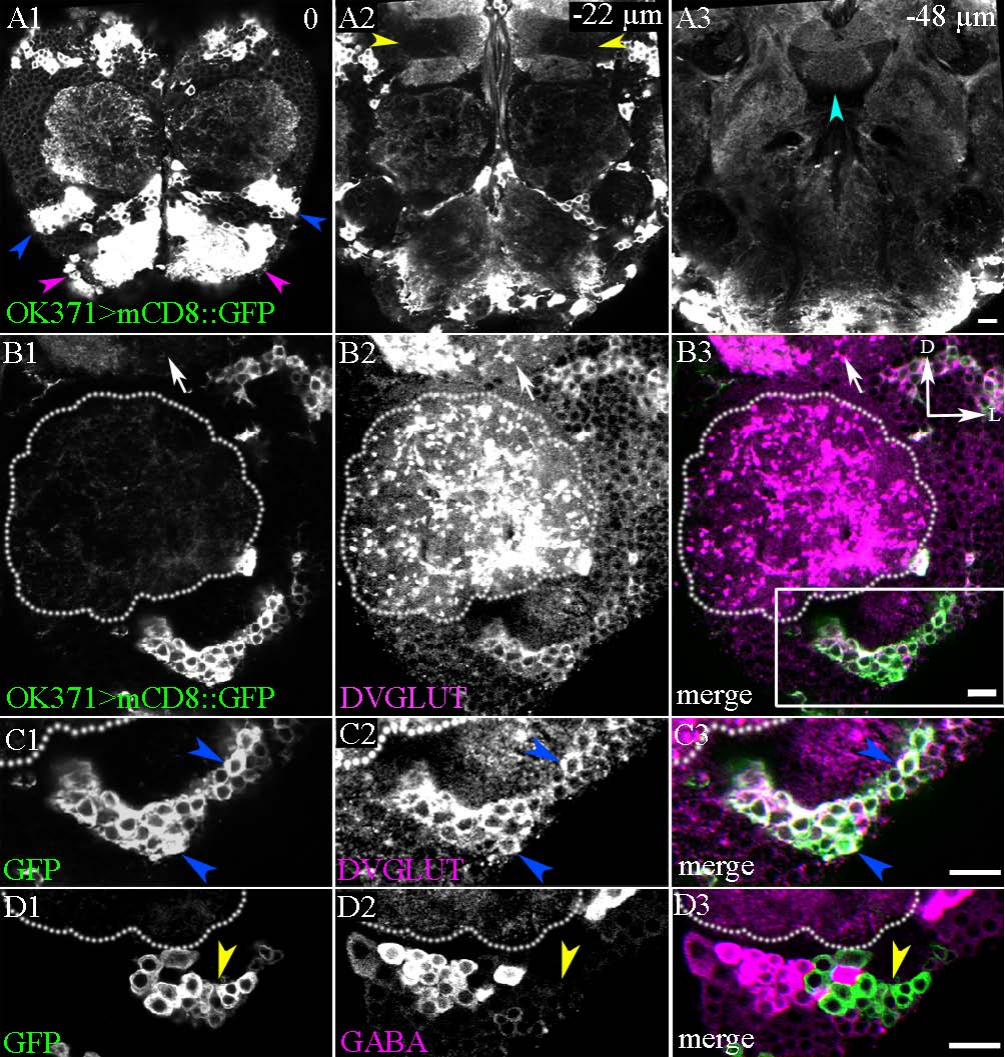
**Gal4-*OK371* labels a ventrolateral glutamatergic local interneuron cluster**. (A) Gal4-*OK371*-driven GFP expression in the adult brain labels clusters of cells that innervate (A1) the antennal lobe (blue arrowheads) and the sub-oesophageal ganglia (magenta arrowheads). (A3) Different neuropil regions are innervated (fan-shaped body, cyan arrowhead) or absent in few neuropil regions; one such example is (A2) the γ lobe of the mushroom body (yellow arrowhead). (B1, D1) Gal4-*OK371*-driven GFP expression labels a cluster of cells located ventrolateral to the adult antennal lobe (demarcated by dotted lines). (B2, B3) These cells are positive for DVGLUT immunoreactivity (magenta). (C) Magnification of the boxed region in (B). (C2, C3) These cells are positive for DVGLUT expression (blue arrowheads). (D1-D3) This cluster of cells is GABA (magenta) negative (yellow arrowhead). Images are oriented as shown in (B3): D = dorsal; L = lateral. Scale bar = 10 μm.

To obtain genetic access to glutamatergic neurons that contribute to olfactory information processing in the antennal lobes, we focused on the enhancer trap line Gal4-*OK371*. The transposable element insertion in this Gal4 line is located ~9 kb upstream of the gene *DVGLUT (CG9887),* which encodes the *Drosophila* vesicular glutamate transporter (DVGLUT), which is required for loading glutamate in the synaptic vesicle [19,22]. Hence, this Gal4 line is likely to be useful for labelling of glutamatergic cells in the fly. For example, this line has been used as a motor neuron marker because it drives reporter gene expression in all glutamatergic motor neurons [19]. Analysis of reporter gene expression (Gal4-*OK371*-driven murine (m)CD8::GFP (green fluorescent protein)) in the adult brain revealed labelled neuronal cell bodies in many diverse cephalic regions (Figure 1A1-1A3) including cells located close to the antennal lobe (Figure 1A1, blue arrowheads; Figure 1B3, white inset). In accordance with a previous report [19], GFP labelling was strongest in the cell bodies of these neurons; GFP expression in their neurites was considerably weaker.

Among the neuronal cell bodies associated with the antennal lobe, we identified a discrete cluster of approximately 65 labelled cells located ventral and slightly lateral to the neuropil of the antennal lobe, to which we refer hereafter as the ventrolateral cluster. Although other Gal4-*OK371*-labelled cells were occasionally observed in the vicinity of this cell cluster in some preparations, this ventrolateral cluster was the only set of cells that was consistently labelled at this spatial location in all preparations. Given the molecular nature of the Gal4-*OK371* enhancer trap line, we considered that the cells in this cluster might be glutamatergic.

To determine if this was the case, we carried out immunolabelling studies with an antibody against the *Drosophila* vesicular glutamate transporter (DVGLUT) [19], together with Gal4-*OK371*-driven mCD8::GFP labelling (Figure 1B1-1B3). Although many of the DVGLUT-immunoreactive cells in the brain were not labelled with the OK371 line (for example, Figure 1B1-1B3, white arrows), the cells of the ventrolateral cluster were consistently DVGLUT-immunopositive. Remarkably, close inspection of the co-labelled preparations indicated that all of the cells in the ventrolateral cluster labelled with the OK371 line were also DVGLUT-immunopositive (Figure 1C1-1C3). We analysed the samples further and counted the number of cell bodies co-labelled with Gal4-*OK371*-driven mCD8::GFP and DVGLUT, which was 62.2 ± 1.4 (mean ± SEM; n = 5). This implies that the ventrolateral cluster is composed exclusively of glutamatergic neurons. Because anatomical analysis indicated that the cells in the ventrolateral cluster are antennal lobe LNs (see below), and because the majority of the previously identified LNs were GABAergic, we performed immunolabelling studies with an anti-GABA antibody together with Gal4-*OK371*-driven mCD8::GFP labelling. Although GABA-immunoreactive cell bodies were observed in close proximity to the OK371-labelled cells of the ventrolateral cluster, none of the OK371-labelled cells was GABA immunopositive (Figure 1D1-1D3). We conclude that all of the cells in the ventrolateral cluster are probably glutamatergic, and none of them is GABAergic. Another enhancer trap line, Gal4-*OK107*, was also found to label a comparable ventral cluster of cells [11] (see Additional file1, Figure S1A), and all cells of this cluster were seen to be DVGLUT immunopositive (see Additional file 1, Figure S1B,C) and GABA immunonegative (see Additional file 1, Figure S1D), thus providing additional confirmation of the findings obtained with Gal4-*OK371*.

### Lineage relationship of the glutamatergic cells in the ventrolateral cluster

**Figure 2:**
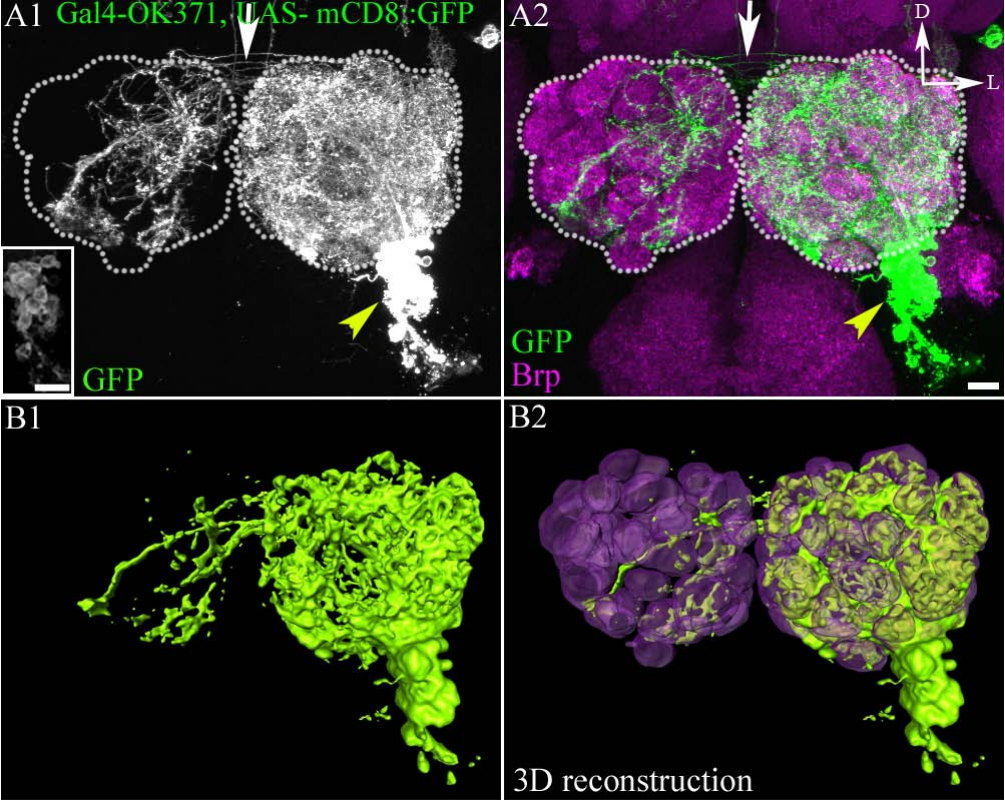
**The ventrolateral glutamatergic cell cluster is born from a single lineage**. (A) MARCM-derived neuroblast clone labelled with Gal4-*OK371* generated by heat shock at 12 ALH. (A1, A2) A cluster of cells (yellow arrowheads) located ventrolateral to the antennal lobe (demarcated by white dots) is labelled. (A2) The antennal glomeruli were labelled with staining with mAbnc82 (Brp) (magenta). (A1 inset) A few confocal sections stacked to enable clearer visualization of somata. (A1, A2) Commissural contralateral projections (indicated by white arrow). (B) Confocal sections were used to reconstruct the interneuron cluster using Amira http://www.amira.com. Scale bar = 10 μm. Images are oriented as shown in (A2): D = dorsal; L = lateral.

Given their clustered nature, we reasoned that all of the cells in the ventrolateral cluster might derive from a single neuroblast. Alternatively, these glutamatergic cells could be produced by two or more neuroblast lineages that are closely apposed in the antennal lobe cell body region. To determine which of these lineage relationships holds for the ventrolateral cell cluster, we used the MARCM clonal analysis method [21] to induce neuroblast clones with Gal4-*OK371* driving a UAS-mCD8::GFP reporter. Clones were induced at larval hatching, and therefore all adult-specific cells labelled with the OK371 line were visualized (note that we only focussed on the labelled cells of the glutamatergic ventral cell cluster; other neighbouring cells in the brain labelled with OK371 were not considered). If the cells of the ventrolateral cluster derive from a single lineage, then all of these (adult-specific) cells should be labelled in all of the MARCM experiments. By contrast, if the cells in the cluster derive from more than one lineage, only a subset of the cells should be labelled in any given MARCM experiment. In all of our experiments, labelled (unilateral) neuroblast clones contained the entire ventrolateral cell cluster, indicating that all cells of the cluster derive from a single neuroblast (Figure 2). In these neuroblast clones, the total number of cell bodies labelled with Gal4-*OK371*-driven mCD8::GFP was 62.9 ± 3.07 (mean ± SEM; n = 8).

This conclusion was supported by results from experiments in which dividing cells were ablated by the DNA-synthesis inhibitor hydroxyurea (HU) [6,12]. The adult antennal lobe of animals, fed with media containing HU at different larval stages, were examined for the presence of the OK371-marked neurons (see Additional file 2, Figure S2). Animals fed with HU at 0-4 hours after larval hatching (ALH), had a normal complement of OK371 cells within the ventrolateral cluster (see Additional file 2: yellow dotted region in Figure S2B compared with Figure S2A). However, HU feeding at 24 hours ALH led to complete ablation of the entire cluster (see Additional file 2, Figure S2C, yellow arrow). This suggests that the neuroblast contributing to this cluster of cells undergoes division at around 24 hours ALH. HU treatment at later time points (36 hours ALH; see Additional file 2, Figure S2D) led to partial ablation of this cluster of interneurons, leaving those cells born between 24 and 36 hours ALH intact (see Additional file 2, Figure S2D, arrow in right hemisphere).

The MARCM-labelled neuroblast clones with Gal4-*OK371*-driven mCD8::GFP also made it possible to visualize the neuroanatomical features of ventrolateral cell cluster neurons in the antennal lobes (Figure 2A). In these labelled clones, a thick neurite bundle emanating from the ventrolateral cell cluster, projects into the ipsilateral antennal lobe, where its neurites branch profusely, resulting in a dense innervation of most of the glomerular neuropil. Some of the neurites cross the midline via a commissural pathway (Figure 2A, white arrow), enter the contralateral antennal lobe, and form arbours, which are, however, not as profuse as the ipsilateral branches and generally remain restricted to the more posterior glomeruli.(the difference in ipsilateral versus contralateral projections is most obvious in 3D reconstructions of the labelled neurites; see Figure 2B1-2B2.) No labelled processes were seen projecting outside of the two antennal lobes.

Taken together, these findings imply that all the glutamatergic neurons of the ventrolateral cell cluster derive from a single neuroblast lineage. Moreover, they indicate that these OK371-labelled cells in this lineage are LNs; none had the anatomical features of PNs. We conclude that the OK371-labelled ventrolateral cluster cells are glutamatergic LNs of a common lineage.

### Glutamatergic local interneurons derive from a novel neuroblast lineage

The experiments mentioned above indicate that all cells of the ventrolateral cluster belong to the same neuroblast lineage. However, they do not indicate whether these cells represent the entire lineage of adult-specific cells, or are only a subset of the cells in a larger lineage that also contains other neuronal cell types. This is a valid consideration, because the glutamatergic ventrolateral cluster is in close spatial proximity to the neuron cluster generated by the ventral lineage. Thus, it is conceivable that the ventrolateral cluster represents a sublineage of the ventral lineage [4].

**Figure 3:**
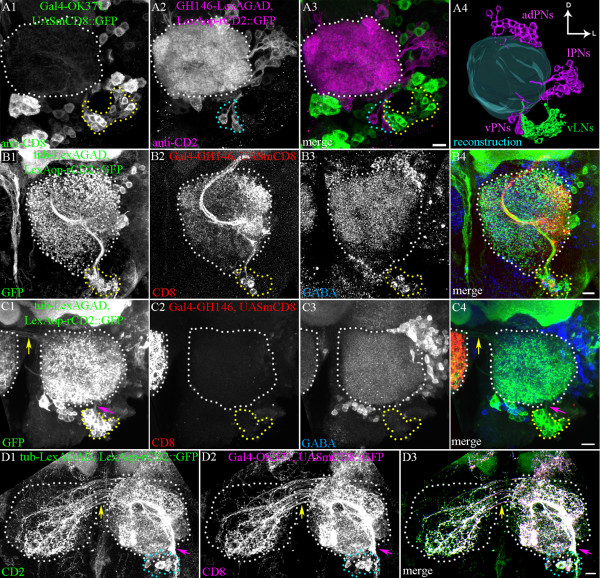
**Local interneurons (LNs) of the glutamatergic ventrolateral cluster derive from a novel lineage**. (A) Dual binary expression system to label (A1) LNs using Gal4-*OK371* > UAS-mCD8::GFP and (A2) projection neurons (PNs) using GH146-LexA::GAD > rCD2::GFP. (A3, A4) A clear separation of the LNs (green) and PNs (magenta) can be seen. (B1-B4) *tub*-LexA::GAD/+ (or Y); FRTG13, Gal4-*GH146*, UAS-mCD8/FRT G13, hsFLP, *tub*-GAL80 ; lexAop-rCD2::GFP/+. Dual MARCM clone induced at 0-4 ALH. Clone labelled with (B1) *Tub* is (demarcated with yellow dots) and (B2) Gal4-*GH146* (yellow dots) is labelled with (B3) anti-GABA; (B4) Merged image of the different channels in B1-B3. (C1-C4) *tub*-LexA::GAD/+ or Y; FRTG13, Gal4-*GH146*, UAS-mCD8/FRT G13, hsFLP, *tub*-GAL80 ; lexAop-rCD2::GFP/+. Dual MARCM clone induced at 0-4 ALH. The clone labelled with (C1) *tub* (yellow dots) is not labelled with (C2) Gal4-*GH146*. (C1, C4) Primary neurite bundle from the cell bodies (magenta arrow). (C3) The clonal cells are not labelled with anti-GABA. (C1, C4) Arbours of these cells cross to the contralateral lobe via the commissure (yellow arrow). (D1-D3) *tub*-LexA::GAD/+ or Y; FRT G13, hsFLP, *tub*-GAL80/UASmCD8::GFP, Gal4-*OK371*, FRTG13; lexAop-rCD2::GFP/+. Dual MARCM clone generated by heat shock at 24 ALH. The clone labelled with (D1, D3) *tub* completely overlaps that labelled with (D2, D3) Gal4-*OK371.* Glutamatergic ventrolateral cluster (cyan dotted line). (A4) Antennal lobes (demarcated by white dots). D = dorsal; L = lateral. Scale bars = 10 μm.

To investigate this, we used two binary gene expression systems to label two cell populations separately. The GAL4-UAS binary system [23] was used to label the ventrolateral cell cluster via expression of mCD8::GFP-driven by Gal4-*OK371,* and in the same preparations, the LexA-based binary system [20] using *GH146*-lexA::GAD to drive rat (r) CD2::GFP reporter expression was used to label PNs of the ventral lineage (vPNs). The GH146 driver labels a subset of the PNs in the ventral, lateral and anterodorsal lineages [4,6]. Subsequent double immunolabelling against CD2 and CD8 allowed selective visualization of the two differentially labelled cell populations. Figure 3A shows that the cell bodies of the ventrolateral cluster are located close to the cell bodies of the vPNs but that the two do not overlap. Although this is an indication that the lineage of the ventrolateral cluster is distinct from that of the ventral vPNs, it does not exclude the possibility of ventrolateral LNs originating from the ventral lineage.

Therefore, to more conclusively demonstrate that the cells of the ventrolateral lineage do not belong to the ventral neuroblast lineage, two sets of dual expression control MARCM experiments were carried out [20]. In first set of experiments, entire neuroblast clones were labelled with a (ubiquitously expressed) *tub*-LexA::GAD-driven rCD2::GFP marker, and Gal4-*GH146*-driven mCD8 was used to assay the presence of vPNs in these clones. Clones were induced between 0 and 24 hours ALH, because the maximum frequency of LN-containing ventrolateral neuroblast clones was generated by recombination events induced during this time.

Two types of neuroblast clones with labelled cell bodies clustered ventrally in the antennal lobe were recovered in adult brains. The first type consisted of approximately 50 cell bodies that projected a bundle into the ipsilateral antennal lobe, where it branched profusely and also gave rise to an axon bundle that exited the lobe (Figure 3B1, 3B4). This axon bundle projected to the lateral horn through the medial antennal cerebral tract (not shown). In these neuroblast clones, *GH146*-driven mCD8 consistently labelled a small set of ventrally located PNs (Figure 3B2, 3B4, red). Moreover, immunolabelling against GABA showed that some of the cells in this neuroblast lineage were GABA-immunoreactive (Figure 3B3, 3B4, blue). Based on these features, these lineages were unambiguously identified as vPN lineages.

The second type of neuroblast clone consisted of a comparable cluster of cells that projected a neurite bundle into the ipsilateral antennal lobe in a similar manner, and formed arbours almost throughout the antennal lobe (Figure 3C1, 3C4). However, unlike the case of the vPN lineage cells, a labelled median antennocerebral tract (mACT) projection was not seen in these neuroblast clones; instead, a labelled commissure (Figure 3C1, 3C4, yellow arrow) was observed, projecting into the contralateral antennal lobe. Moreover, this type of neuroblast clone did not contain any Gal4*-GH146*-driven mCD8-labelled PNs (Figure 3C2), nor were any of its cells GABA immunopositive (Figure 3C3). Hence, this type of neuroblast clone had key cellular and molecular features of the *OK371*-labelled ventrolateral LN cluster and none of the defining features of the vPN lineage, establishing the ventrolateral glutamatergic lineage as distinct from the vPN lineage. We conclude that the ventrolaterally located glutamatergic lineage is a novel lineage that comprises a large population of antennal lobe LNs. From here, we refer to this novel olfactory interneuron lineage as the ventrolateral local interneuron (vlLN) lineage.

In a final set of dual-expression control MARCM experiments, we set out to determine whether all of the (adult-specific) neurons in this novel ventrolateral lineage were labelled with OK371, implying that the entire lineage is composed of glutamatergic LNs, or whether other types of neurons are also present in the lineage. For this, we used the Gal4-*OK371* driver to mark the vlLN population with mCD8, and labelled the entire neuroblast lineage with *tub*-LexA::GAD driving rCD2::GFP. Clones were induced at similar time points (24 hours ALH) and recovered from the adult brains. Remarkably, in all cases there was a virtually complete overlap of the two differentially labelled clones; all cells marked by *tub*-driven CD2 were also marked by OK371-driven CD8 (Figure 3D1-3D3). This finding indicates that the OK371 driver line labels all (adult-specific) cells in this novel lineage. This, in turn, suggests that the entire ventrolateral neuroblast lineage gives rise exclusively to LNs, all of which are apparently glutamatergic.

### Neuroanatomical diversity of the local interneurons in the ventrolateral lineage

**Figure 4:**
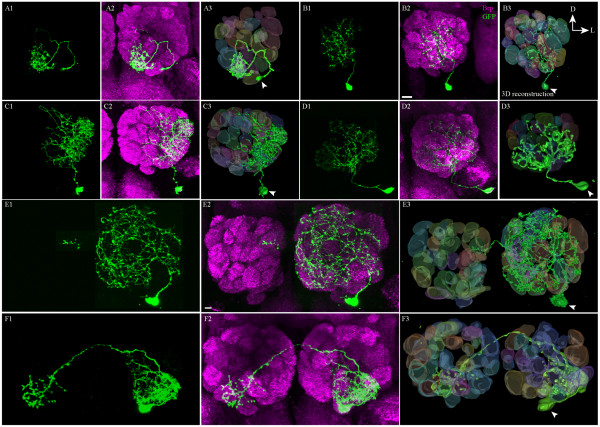
**Diverse neuronal architectures of the ventral local interneurons (vILNs)**. Single cell vlLN clones showing examples of (A-D) ipsilaterally and (E-F) bilaterally projecting neurons. (A) An ipsilaterally innervating oligoglomerular neuron (arborizing in one to three glomeruli). (B-D) Ipsilaterally innervating multiglomerular (arborizing in a large subset of glomeruli) neurons. (A-D) Arbours terminate in contiguous glomeruli (continuous innervation). (E) Bilaterally innervating LN. The innervation is multiglomerular on the ipsilateral side with only oligoglomerular (one to three) innervation contralaterally. (F) Bilaterally innervating neuron with oligoglomerular arbours in both antennal lobes. (A1, B1, C1, D1, E1 F1) Neuron labelled with the mCD8::GFP reporter. (C1, D1, E1, F1) Tiled images made from cropped portions of appropriate sections to highlight the single cell. (A2, B2, C2, D2, E2, F2) Merged images (of GFP and Brp channels) that are reconstructed (A3, B3, C3, D3, E3 and F3) of confocal sections through the antennal lobe using Amira. White arrowheads indicate the cell bodies. Images are oriented as shown in (B3): D = dorsal, L = lateral. Scale bars = 10 μm.

The MARCM method can be used to label single neurons in a given lineage at specific developmental times, and this was used by Chou *et al*. in a descriptive analysis of the morphological types of LNs that innervate the antennal lobe [11]. In a comparable approach, we used single-cell MARCM techniques to visualize individual vlLNs, and subsequently classified them into neuroanatomical types, based on the previously established terminology [11]. We analysed 51 single cells labelled with generating clones at different times during larval life. The results of this analysis are summarized in Table 1 and representative examples of LN morphological types are shown in Figure 4.

**Table 1:** Description of all single cell clones: different neuroanatomical types born at different time points.

**Heat shock time, hours ALH**^**2**^	**Ipsilateral LNs**^**1**^		Bilateral LNs			
			
			Ipsilateral side		Contralateral side	
	
	Oligo- glomerular	Multi- glomerular	Oligo- glomerular	Multi- glomerular	Oligo- glomerular	Multi- glomerular
24	1	-	-	-	-	-
36	-	3	-	1	1	-
48	4	1	-	-	-	-
60	-	1	-	-	-	-
72	2	2	-	-	-	-
84	1	-	1	-	1	-
96	6	4	4	2	6	-
120	5	1	9	3	12	-
Total (n = 51)	19	12	14	6	-	-

^1^LN = local neuron; ^2^ALH = after larval hatching.

Classification of LNs is modified from Chou et al [11]. A total of 51 single cell clones were analyzed, of which 31 were ipsilateral and 20 were bilateral. Each of these types can have either an oligo-, multi- or pan-glomerular innervation pattern, which can be continuous or patchy. Of 51 cells analyzed, all had a continuous innervation pattern and were either oligo- or multi-glomerular, and none was pan-glomerular.

LNs are classified as ipsilaterally and bilaterally arborizing neuron types, and both of these categories are further subdivided based on the extent of arborization within the antennal lobe, grouping them as oligoglomerular (one to three glomeruli innervated), multiglomerular (more than three but not the majority of glomeruli innervated) or panglomerular (all or almost all glomeruli innervated). In addition, the innervation patterns of the oligoglomerular and multiglomerular types were classified as continuous or patchy (for details of the LN classification system, see [11]).

Of the 51 single cells analysed, 31 cells were ipsilaterally and 20 cells were bilaterally projecting LNs; all had a continuous type of innervation pattern. Pan-glomerular LNs were not observed. Two types of ipsilaterally projecting LNs were seen: oligoglomerular (Table 1; example in Figure 4A) and multiglomerular (Figure 4B-4D). Two types of bilaterally projecting LNs were also observed: one was multiglomerular on the ipsilateral side and oligoglomerular on the contralateral side (Figure 4E), and the other type was oligoglomerular on both the ipsilateral and contralateral sides (Figure 4F). No obvious indication for the selective generation of specific LN types during a defined postembryonic developmental period was found; all types were generated throughout postembryonic neurogenesis (Table 1). We further analysed the individual glomerular innervation patterns of each of these single cell clones (see Additional file 3, supplementary table S1). We conclude that the ventrolateral lineage gives rise to a morphologically diverse set of olfactory LNs.

### Postembryonic development of the olfactory local interneurons in the vlLN lineage

**Figure 5:**
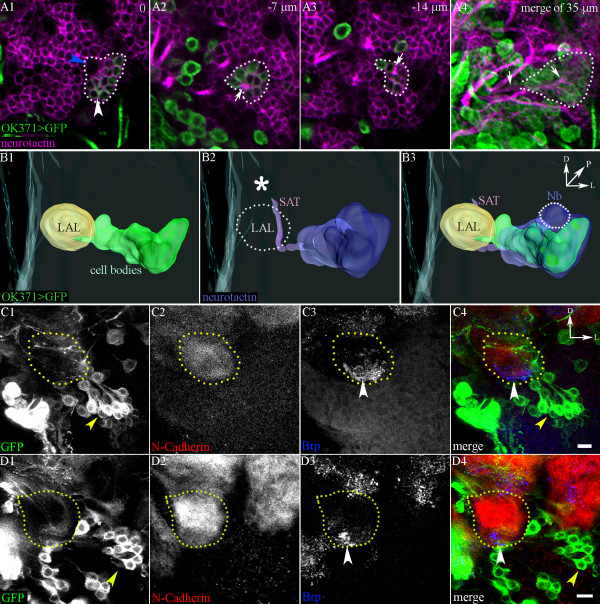
**Developmental profiling of the glutamatergic ventrolateral cluster**. (A) Labelling of developing (third instar larval stage) ventral local interneurons (vILNs) with antibodies against neurotactin (Nrt; magenta). (A1-A3) Sections at 7 μm intervals focus on the cell somata of vlLNs labelled with OK371 (demarcated by white dots). (A1) Neuroblast is not labelled with green fluorescent protein (GFP) (blue arrowhead); GFP co-localizes with neurotactin (Nrt) (white arrowhead), and secondary axonal tract is labelled with anti-Nrt (small white arrows). (A4) Merged image of all 35 confocal sections showing the entire ventrolateral lineage. (B) Reconstruction of appropriate confocal sections using Amira. (B1) Larval antennal lobe (LAL) with ventrolaterally located cell bodies labelled with GFP expression in Gal4-*OK371*. (B2) Reconstruction of the anti-Nrt labelled lineage; the secondary axonal tract (SAT) traverses the LAL (outlined with dots) and terminates near the prospective adult AL (asterisk). (B3) Merge of the images shown in (B1) and (B2); white dotted line demarcates the location of the neuroblast. (C,D) Antennal lobes from (C) 6 and (D) 12 hours APF, showing developing neurons labelled with Gal4-*OK371* (yellow arrowheads). (C2. D2). The presumptive adult antennal lobe is labelled with anti-N-Cadherin (yellow dotted lines). (C3, D3)The regressing larval antennal lobe is identified by staining with mAbnc82 (anti-Brp) (white arrowheads). (D1) Tiled image made from cropped portions of appropriate sections to highlight the cells that are part of this cluster. Orientation of the images; D = dorsal; L = lateral; P = posterior. Scale bar = 10 μm.

To characterize the developing interneurons of the vlLN lineage, we analysed third instar larval brains labelled with mCD8::GFP-driven by Gal4-*OK371* and immunostained with anti-neurotactin, which labels all developing adult-specific neurons and their secondary axonal tracts (Figure 5A, B) [24]. In third instar larval brains, OK371 labelled the entire vlLN cluster, which was, as expected, neurotactin-positive, indicating that these are indeed adult-specific secondary neurons (Figure 5A). (OK371 also labelled some other cells in the vicinity of the ventrolateral cluster; however, these cells were more scattered, larger in size, and clearly separated from the other cells, and were not included in our analysis).

A 3D reconstruction of the labelled developing ventrolateral cluster confirmed the position of the developing lineage relative to the larval antennal lobe (Figure 5B). Moreover, it allowed visualization of the neurotactin-positive secondary axonal tract (SAT) (Figure 5A, white arrows), which emanates from the cluster, projects through the region of the larval antennal lobe (LAL) and then turns anteriorly to reach the region of prospective adult antennal lobe (Figure 5B2, white asterisk), where it terminates abruptly. Although the neuroblast that gives rise to the ventrolateral lineage is not labelled with OK371, this progenitor cell is visualized by anti-neurotactin staining and can be identified based on its position relative to the labelled cluster (Figure 5A1, blue arrowhead; Figure 5B3, cluster is demarcated by white dots).

Because secondary, adult-specific neurons are generated during larval stages and generally differentiate during metamorphosis, we followed the development of the ventrolateral lineage into the early pupal stages. In these experiments, the developing adult antennal lobe primordium was labelled with anti-N-cadherin (Figure 5C, 5D, demarcated by yellow dots) and the regressing larval antennal lobe was labelled with anti-Brp (Figure 5C3, 5D3, arrowheads). As early as 6 hours after puparium formation (APF), axonal projections from the ventrolateral cluster make contact with the adult antennal lobe primordium (Figure 5C). By 12 hours APF, these axons grow around the remnants of the larval antennal lobe and begin to invade the developing adult lobe (Figure 5D). Comparable findings were obtained when the 'enhancer-trap' line Gal4-*OK107* was used to label the ventrolateral cluster (see Additional file 4, Figure S3A,B). DVGLUT immunoreactivity, which presumably marks the terminals of the vlLNs, is first observed in the developing adult antennal lobe at 12 hours APF (see Additional file , Figure S3B3, cyan arrowhead) and increases during later pupal life (see Additional file 4, Figure S3C3, D3) indicating the formation of additional arbours.

**Figure 6:**
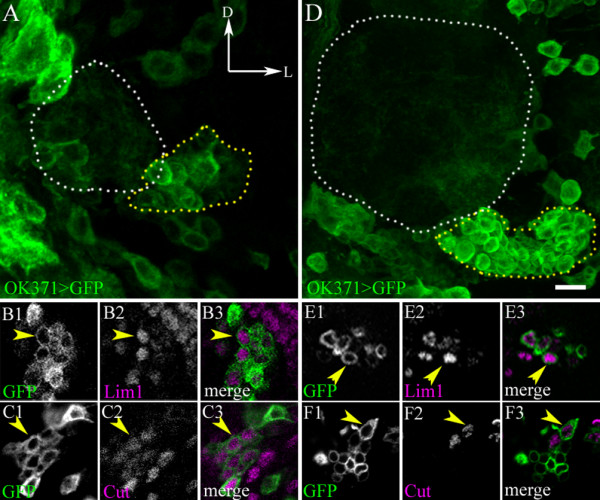
**Transcription factor expression in the ventrolateral lineage interneurons (vILNs)**. Third instar (A-C) larval and (D-F) adult brains with vlLNs labelled with Gal4-*OK371*, UAS-mCD8::GFP. (A,D) Antennal lobe (white dots) and neurons (yellow dots) in the lineage. (B, C, E, F) Single confocal sections through the somata from (B,C) larval and (E,F) adult brains labelled with antibodies against (B,E) dLim1 and (C,F) Cut. Labelling is more abundant in developing than in adult brains. (B, C, E and F) Cell bodies co-labelled with the corresponding transcription factor (yellow arrowheads). D = dorsal, L = lateral. Scale bar = 10 μm.

Antennal lobe interneurons derived from the three well-studied lineages are known to express a set of transcription factors, which play key roles in axonal pathfinding and targeting. Among these are the Acj6, dLim1 and Cut [25]. To determine whether these molecules are also expressed in the developing interneurons of the ventrolateral lineage, we studied the expression of Acj6, dLim1 and Cut in immunocytochemical experiments, together with OK371 labelling. Acj6, which is known to be expressed in anterodorsal PNs and some atypical PNs of the lateral lineage during postembryonic development [5,26], was not expressed in the developing ventrolateral lineage (data not shown). By contrast, dLim1, which is expressed by most of the lateral LNs and ventral PNs during larval development [25,27], was also expressed in the vlLNs (Figure 6). During postembryonic development, dLim1 was expressed in all of the cells in this lineage (Figure 6B), and then became restricted to a small number of vlLNs in the adult (Figure 6E). The homeodomain protein, Cut, which is expressed during the development of all three previously characterized olfactory interneuron lineages, was also expressed in the vlLNs at the larval stage (Figure 6C), and became restricted to a few cells in the adult (Figure 6F). Similar results were observed when the vlLN cluster was labelled with the Gal4-*OK107* line (see Additional file 5, Figure S4).

Taken together, these observations reveal several similarities between the developing neurons of the ventrolateral lineage and the developing neurons of the other three antennal lobe interneuron lineages. In all four lineages, the adult-specific neurons are generated during larval stages, and form secondary axon tracts that project towards the adult antennal lobe primordium. Moreover, in all four lineages, innervation of the developing adult antennal lobe occurs during metamorphosis in pupal stages. Finally, at least in the case of dLim1 and Cut, all four lineages express a common subset of developmentally relevant transcription factors.

## Discussion

All the central neurons in the *Drosophila* antennal lobe are thought to be derived from three neuroblast lineages, two of which (anterodorsal and ventral lineages) give rise to a large set of diverse PNs [4]. The third (lateral) lineage also generates PNs, but in addition, it gives rise to olfactory LNs, which are largely inhibitory (GABAergic) with a few excitatory (cholinergic) cells [5,12]. A recent study on the diversity of olfactory LNs has provided evidence for the presence of glutamatergic LNs [11], and we built up the tentative observation of these glutamatergic LNs as a distinct cluster. In this paper, we show that these neurons arise from a novel, hitherto unidentified neuroblast, the ventrolateral neuroblast.

We used MARCM-based clonal analysis to describe the anatomical features of the LNs arising from the ventrolateral neuroblast. Although the vlLNs resemble the lateral LNs in their structure, they are distinct in their glutamatergic neurotransmitter identity. The lineage of this 'fourth' neuroblast differs from the three previously studied interneuron lineages in that it is exclusively composed of LNs, unlike the other neuroblasts, which also give rise to PNs. The absence of PNs in this lineage may be responsible for its delayed identification relative to the other interneuron lineages. The anterodorsal, lateral and ventral clusters of cells were initially identified based on the anatomical localization of the PN somata. These and their axonal projections in specific brain tracts are labelled with the Gal4-*GH146* strain, which labels a subset of PNs in each of the three lineages but does not label LNs. Our initial identification of the ventrolateral lineage was facilitated by expression of the Gal4-*OK371* line. The ventrolateral lineage, like the other three antennal lobe lineages, can now be genetically accessed, and this should facilitate further comprehensive studies of function and development of olfactory circuitry in the antennal lobe.

Although all of the cells in the ventrolateral lineage are LNs, they nevertheless represent a structurally diverse group in terms of their individual glomerular innervation patterns. Our single cell analysis revealed both cells with ipsilateral and cells with bilateral glomerular arborizations. These arbours can be oligoglomerular (which are restricted to a small number of glomeruli (one to three)) or multiglomerular, with innervation in a larger number of glomeruli. Interestingly, we did not observe LNs with panglomerular innervation patterns. This, together with their excitatory (glutamatergic) neurotransmitter phenotype, implies that the functional roles of these LNs is in mediating specific excitatory interactions between subsets of glomeruli rather than in distributing excitatory activity to all (or most) of the glomeruli in the antennal lobe. This notion is in accordance with neurophysiological findings that reveal the existence of olfactory circuit elements that mediate stereotyped selective local, but not global, excitatory interconnections in the antennal lobe glomeruli [13,14].

Although they are individually selective in their specific innervation patterns and hence, in their distribution of excitation among glomeruli, the overall influence of these LNs is likely to be pronounced. This is because an entire neuroblast lineage, comprising ~70 cells, appears to be dedicated to mediating excitatory interactions in the antennal lobe. This extensive excitatory LN network might be the neuroanatomical substrate that mediates the pervasive excitatory functional connections between olfactory glomeruli, which have been shown to be very dense and probably involve excitatory interactions of an 'all to all' nature [13]. Indeed, the ventrolateral LN network may play a central role in distributing the excitatory olfactory information carried by the glomerulus-specific axons projections of each OSN type to all of the glomeruli in the antennal lobe.

The observation that all ventrolateral LNs have the same neurotransmitter is interesting in terms of a general lineage-based modular organization of the fly brain. Thus, brain neuroblast lineages might not only comprise modules with similar developmental genetic and neuroanatomical features but also correspond to modules with comparable biochemical (neurotransmitter) features. In this respect, it will be interesting to investigate whether other neuroblasts in the brain generate neuronal lineages or sublineages that have the same neurotransmitter phenotype. A detailed knowledge of the lineages of the four lobe neuroblasts offers a system for investigating the role of combinatorial ensembles of transcription factors (dLim1, Cut and other unidentified transcription factors) in lineage specification, wiring specificity (dendrite/axon targeting) and neurotransmitter identity [25,26,28-31].

## Conclusions

The identification of a novel fourth neuroblast lineage of olfactory neurons in the antennal lobe of *Drosophila* makes an important contribution to a more complete understanding of the development and organization of this model neural circuitry. Moreover, it reveals an unexpected homogeneity in three features of the neural components of this lineage. Thus, our data imply that all cells in this lineage are local interneurons, all have a glutamatergic neurotransmitter phenotype, and all form oligoglomerular or multiglomerular innervation of the antennal lobe. The identification of this novel lineage and the elucidation of the innervation patterns of its local interneurons (at single cell resolution) provides a comprehensive framework for a systematic analysis of the cellular and molecular determinants that mediate the development and interconnectivity of glutamatergic excitatory local interneurons in the circuits of the *Drosophila* antennal lobe.

## Methods

### Fly stocks and genetics

All flies were raised at 25°C on standard cornmeal agar medium unless otherwise specified. Gal4-*OK371* and Gal4-*OK107* were obtained from Hermann Aberle (University of Muenster, Germany) and Cahir O'Kane (University of Cambridge, UK), respectively. All stocks used for dual expression control MARCM experiments (*y*,*w*, *tub*-LexA::GAD; *Pin/CyO,y*+, *y*,*w*; FRTG13, hsFLP, *tub*-GAL80/*CyO, y*+, *y*, *w*; FRTG13, UAS-mCD8, lex-Aop-rCD2::GFP/*CyO, y*+, *y*, *w*; FRTG13, Gal4-*GH146*, UAS-mCD8/*CyO, y*+, *y*, *w*; *Pin/CyO*, *y*+; lexAop-rCD2::GFP) were kindly provided by Tzumin Lee (Janelia Farm Research Campus, HHMI, USA) and *y*,*w*; FRT G13, UAS-mCD8::GFP, OK371-Gal4/Cyo was provided by Kirti Rathore (National Centre for Biological Sciences, Bangalore, India). All other stocks were obtained from the Bloomington *Drosophila* Stock Centre, Indiana University, USA.

### MARCM and dual expression control MARCM experiments

The MARCM system [21] was used to generate mCD8::GFP-labeled clones. To generate Gal4-*OK371* labelled clones, females of hs-flp, UASmCD8::GFP; FRTG13, *tub*-GAL80/*CyO* were crossed to male UASmCD8::GFP, Gal4-*OK371*, FRTG13/*CyO* flies. To generate dual MARCM clones, females of *tub*-LexA::GAD; FRT G13, hsFLP, *tub*-GAL80/*CyO, y*+ were crossed to males of either UASmCD8::GFP, Gal4-*OK371*, FRTG13/*CyO*, lexAop-rCD2::GFP/TM6B (for *tub*-*OK371* Dual MARCM) or *y*,*w*/*Y*; FRTG13, Gal4-*GH146*, UAS-mCD8/*CyO, y*+; lexAop-rCD2::GFP (for *tub-GH146* Dual MARCM). Embryos from the above crosses were collected at 4 hours intervals and reared at 25°C. Heat shocks were given at the required time points for 1 hour in a water bath maintained at 37°C. Cultures were then returned to 25°C, and animals were allowed to develop to adulthood.

### Immunohistochemistry

Dissection of adult brains were carried out in 1X phosphate-buffered saline (PBS) and fixed in 4% freshly prepared PFA (prepared in 1× PBS) for 30 minutes at room temperature. The fixative was removed, and the brains were washed four times with 0.3% PTX (0.3% Triton X-100 in 1× PBS ) for 15 minutes each at room temperature. Blocking of samples was performed for 30 minutes at room temperature in 5% normal goat serum (NGS, cat#NS1, Genei, Bangalore, India) prepared in 0.3% PTX. Primary antibodies used were rabbit anti-GFP (1:10,000; Molecular Probes, Invitrogen, USA), chick anti-GFP (1:500; Abcam, Cambridge, UK), rat anti-mCD8 (1:100; Caltag Laboratories, Burlingame, CA, USA), mouse anti-rCD2 (1:100; Serotec, Raleigh, NC, USA), mouse anti-Bruchpilot (Brp) (mAbnc82, 1:20; DSHB, Iowa, USA), mouse anti-neurotactin (Nrt) (BP106, 1:10; DSHB), mouse anti-Cut (2B10, 1:50; DSHB), rabbit anti-GABA (1:500; cat#A2052, Sigma, St Louis, MO, USA), rabbit anti-C-term DVGLUT (1:500; a gift from Hermann Aberle and Aaron DiAntonio (Washington University in St. Louis, MO, USA)) and rabbit anti-d-Lim1 (1:1000; a gift from Juan Botas (Baylor College of Medicine, TX, USA)) [32].

For labelling with anti-Brp and anti-DVGLUT, samples were incubated at 4°C for 48 hours. They were then washed in 0.5% PTX and then incubated overnight in secondary antibodies in 4°C. Secondary antibodies used were Alexa-488, Alexa-568 and Alexa-647 coupled antibodies generated in goat (Molecular Probes), all used at 1:400 dilutions. After washing, labelled samples were mounted in 70% glycerol and viewed under a confocal microscope (Fluoview FV1000 Laser Scanning Confocal Microscope; Olympus, Tokyo, Japan) at 1 μm intervals with a picture size of 512 × 512 pixels. Data were processed using ImageJ software (http://rsbweb.nih.gov/ij/ and pseudo-colouring, and enhancements were performed with Adobe Photoshop CS3 (Adobe Systems Incorporated, CA, 1USA). The 3D reconstructions were generated with the Amira 5.2.0 software (Visage Imaging, Berlin, Germany). Figures were prepared as previously described http://jfly.iam.u-tokyo.ac.jp/html/color_blind/_.

## Competing interests

The authors declare that they have no competing interests.

## Authors' contributions

AD, AC, HR, KVR and VR conceptualized the project, planned the experiments, and wrote the manuscript. AD, AC, SD and RP carried out the experiments and analyzed the data. All authors have read the manuscript and agree with the contents.

## Acknowledgements

We thank Cahir O'Kane, Hermann Aberle, Reinhard Stocker, Tzumin Lee, Liqun Luo, Kirti Rathore and the Bloomington *Drosophila* Stock Centre for fly stocks, Aaron DiAntonio, Hermann Aberle, Juan Botas and the Developmental Studies Hybridoma Bank (Iowa, USA) for antibodies. This work was supported by grants from TIFR and the Indo-Swiss Joint Research Program. A.C. is a Wellcome Trust- DBT India Alliance Early Career Fellow. We thank the Department of Science and Technology, Government of India-(Centre for Nanotechnology Number SR/S5/NM-36/2005) for the Olympus FV1000 confocal microscopes in the Central Imaging and Flow Cytometry Facility (CIFF) and NOMIC, NCBS.

Veronica Rodrigues, our colleague and friend died after a 5- year struggle with cancer. To the end, she was an inspiration to us, as she always was to her many students and collaborators over the years. We remember her fondly and our continued collaborations will be testimony to her deep influence.

## Supplementary Material

Additional file 1 **Supplemental Figure S1: The 'enhancer-trap' line Gal4-*OK107* labels the ventrolateral cluster of local interneurons**. Gal4-*OK107* driving mCD8::GFP. Adult brains labelled with (A) mAbnc82 (anti-Brp); (B, C) anti-DVGLUT; (D) anti-GABA. (A, A3) The cluster of cells (yellow arrowhead) send neurites into the antennal lobe. (C, D) Enlargement of the 'boxed-in' area in B3, showing expression of (C) DVGLUT (blue arrowheads) and (D) no expression of GABA (yellow arrowheads). D = dorsal, L = Lateral. Scale bar = 10 μm. Click here for file

Additional file 2 **Supplemental Figure S2: Hydroxyurea (HU)-mediated ablation of ventrolateral lineage**. Adult brains of Gal4-*OK371*>UAS-mCD8::GFP stained with antibodies against green fluorescent protein (GFP) (green) and mAbnc82 (anti-Brp) (magenta). The antennal lobes are demarcated with white dots and the cells of the cluster with yellow dots. (A) Control; (B-D) brains from flies fed on an HU-containing diet at (B) 0-4 hours ALH, (C) 24 hours ALH and (D) 36 hours ALH. (C, D) Ventrolateral cluster (yellow arrows). Scale bar = 10 μm. Click here for file

Additional file 3 Supplementary Table S1: Glomerular innervation pattern of both ipsilateral and bilateral LNs. Click here for file

Additional file 4 **Supplemental Figure S3: Developmental profile of ventrolateral cluster neurons labelled with Gal4-*OK107***. Gal4-*OK107*>UASmCD8::GFP antennal lobes (demarcated with white dots) at (A) 6, (B) 12, (C) 48 and (D) 72 hours after puparium formation (APF). Ventrolateral cluster neurons are labelled with yellow arrowhead. (A2, B2, C2, D2) The antennal lobe is labelled with anti-N-cadherin. Twelve hours APF, (B3) there are sparse *Drosophila* vesicular glutamate transporter (DVGLUT). immunopositive punctae that are concomitant with (B1, B4) GFP-labelled fibre innervation. (C3, D3) DVGLUT labels more extensively within the lobe neuropil. (A1, B1) Tiled images made from cropped portions of appropriate sections to highlight the cells that are part of this cluster. Scale bar = 10 μm. D = dorsal, L = lateral. Click here for file

Additional file 5 **Supplemental Figure S4: Expression of dLim1 and Cut in the ventrolateral lineage interneurons labelled with OK107**. Third instar larval (A-C) and adult (D-F) brains with vlLNs labelled with Gal4-*OK107*, UAS-mCD8::GFP. (A,D) Antennal lobe is demarcated with white dots and the neurons in the lineage by yellow dots. (B, C, E, F) Enlarged regions of cells from (B, C) larval and (E,F) adult brains labelled with antibodies against (B, E) dLim1 and (C,F) Cut. Scale bar = 10 μm. D = dorsal, L = lateral. Click here for file
