# Supplementary figures and images for: Identification and analysis of a glutamatergic local interneuron lineage in the adult Drosophila olfactory system
Source: Neural Syst Circuits. 2011 Jan 26;1:4. doi: 10.1186/2042-1001-1-4 (PMC3257541; doi:10.1186/2042-1001-1-4)

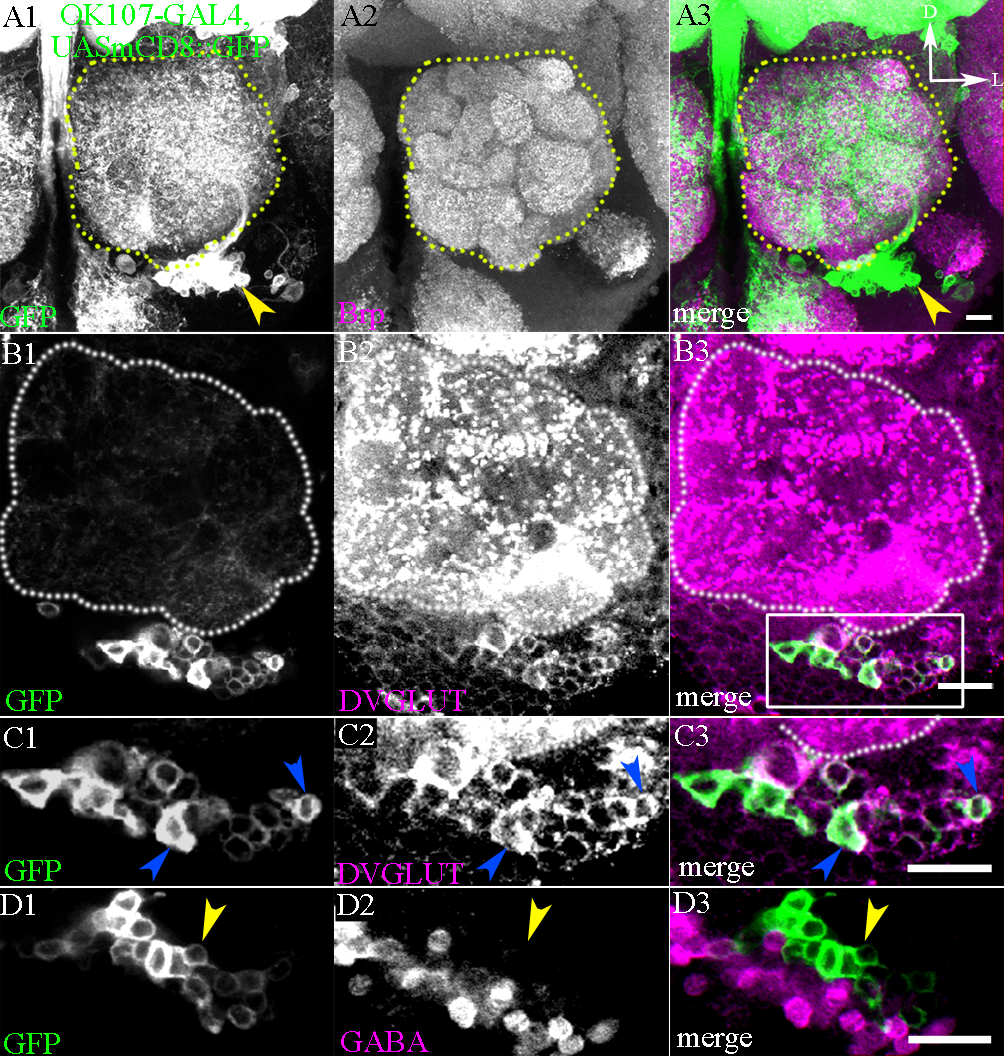

Supplement: Additional file 1 — Supplemental Figure S1: The 'enhancer-trap' line Gal4-OK107 labels the ventrolateral cluster of local interneurons. Gal4-OK107 driving mCD8::GFP. Adult brains labelled with (A) mAbnc82 (anti-Brp); (B, C) anti-DVGLUT; (D) anti-GABA. (A, A3) The cluster of cells (yellow arrowhead) send neurites into the antennal lobe. (C, D) Enlargement of the 'boxed-in' area in B3, showing expression of (C) DVGLUT (blue arrowheads) and (D) no expression of GABA (yellow arrowheads). D = dorsal, L = Lateral. Scale bar = 10 μm. [file 2042-1001-1-4-S1.TIFF]

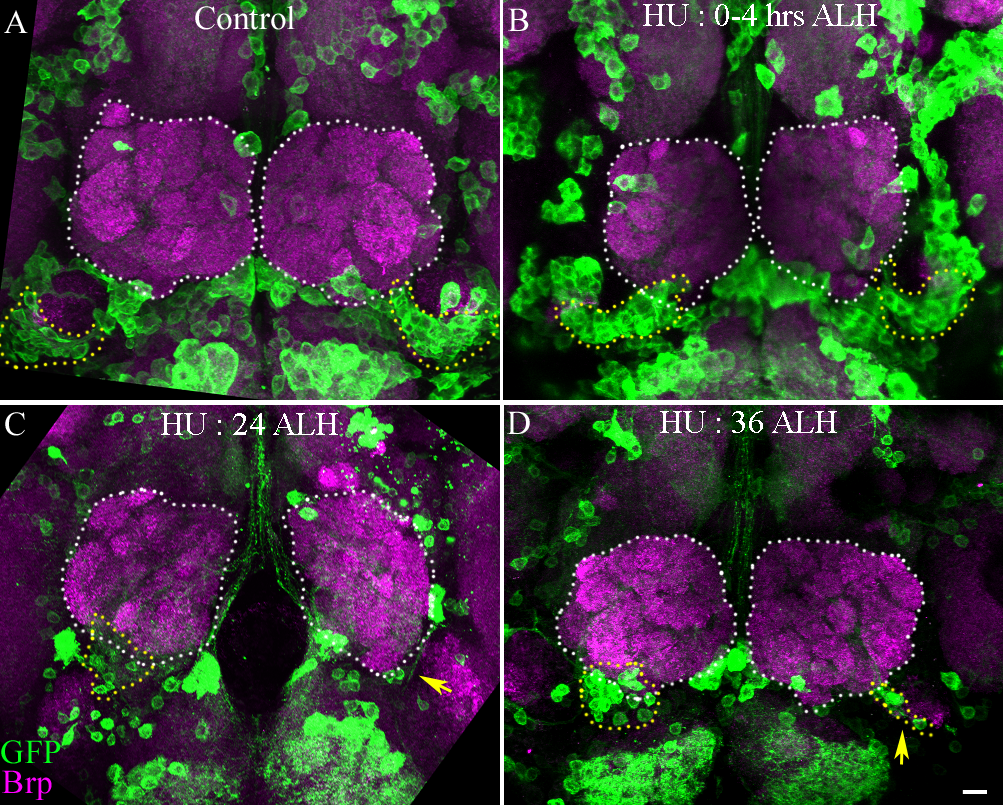

Supplement: Additional file 2 — Supplemental Figure S2: Hydroxyurea (HU)-mediated ablation of ventrolateral lineage. Adult brains of Gal4-OK371>UAS-mCD8::GFP stained with antibodies against green fluorescent protein (GFP) (green) and mAbnc82 (anti-Brp) (magenta). The antennal lobes are demarcated with white dots and the cells of the cluster with yellow dots. (A) Control; (B-D) brains from flies fed on an HU-containing diet at (B) 0-4 hours ALH, (C) 24 hours ALH and (D) 36 hours ALH. (C, D) Ventrolateral cluster (yellow arrows). Scale bar = 10 μm. [file 2042-1001-1-4-S2.TIFF]

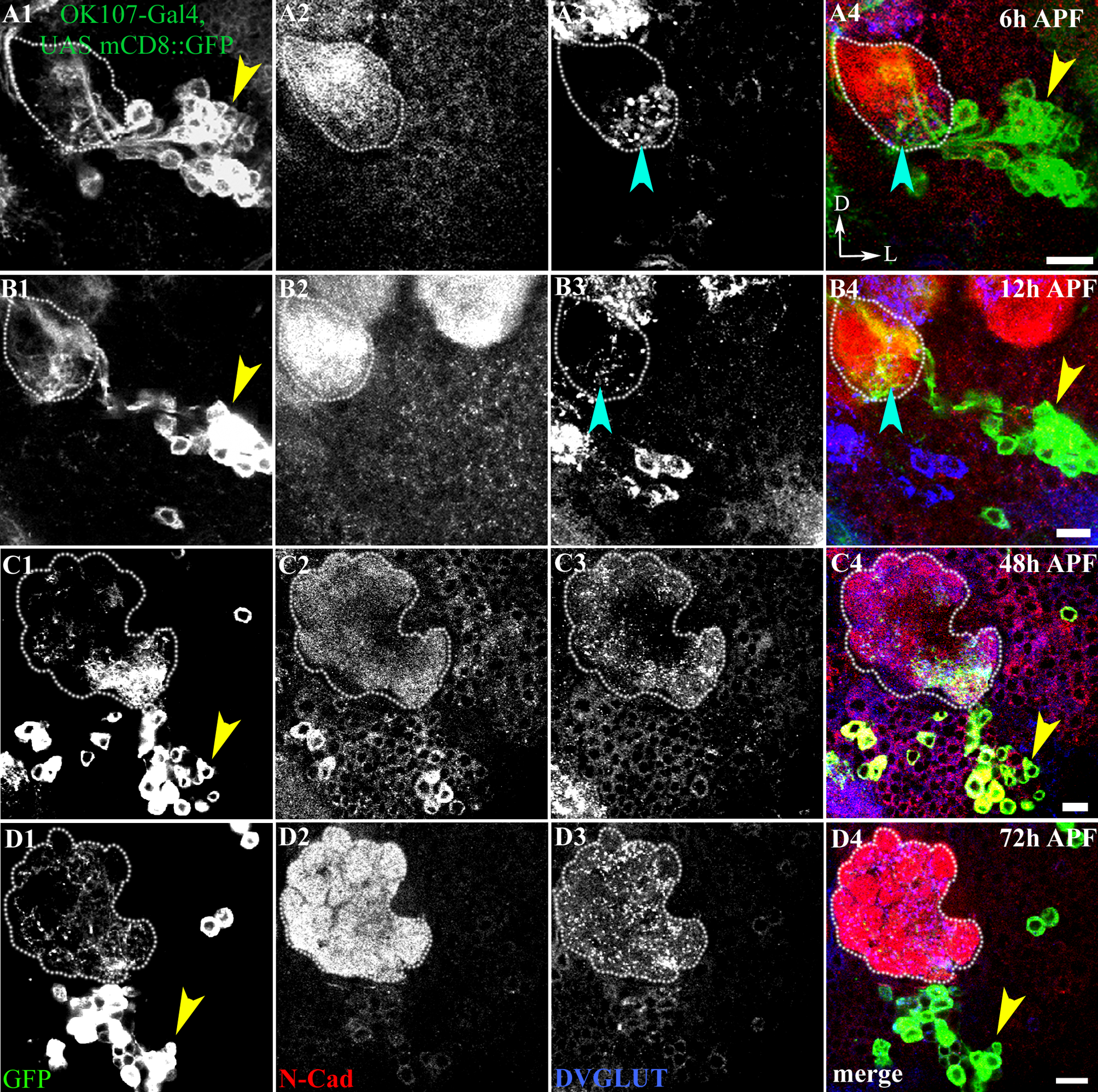

Supplement: Additional file 4 — Supplemental Figure S3: Developmental profile of ventrolateral cluster neurons labelled with Gal4-OK107. Gal4-OK107>UASmCD8::GFP antennal lobes (demarcated with white dots) at (A) 6, (B) 12, (C) 48 and (D) 72 hours after puparium formation (APF). Ventrolateral cluster neurons are labelled with yellow arrowhead. (A2, B2, C2, D2) The antennal lobe is labelled with anti-N-cadherin. Twelve hours APF, (B3) there are sparse Drosophila vesicular glutamate transporter (DVGLUT). immunopositive punctae that are concomitant with (B1, B4) GFP-labelled fibre innervation. (C3, D3) DVGLUT labels more extensively within the lobe neuropil. (A1, B1) Tiled images made from cropped portions of appropriate sections to highlight the cells that are part of this cluster. Scale bar = 10 μm. D = dorsal, L = lateral. [file 2042-1001-1-4-S4.TIFF]

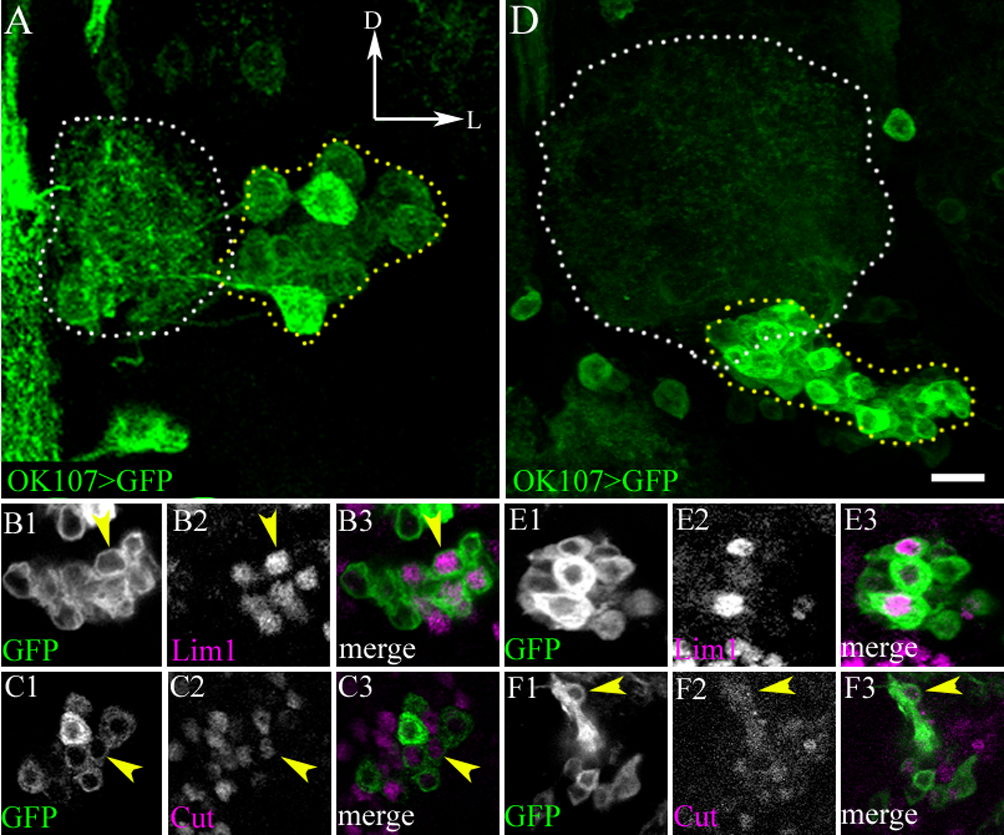

Supplement: Additional file 5 — Supplemental Figure S4: Expression of dLim1 and Cut in the ventrolateral lineage interneurons labelled with OK107. Third instar larval (A-C) and adult (D-F) brains with vlLNs labelled with Gal4-OK107, UAS-mCD8::GFP. (A,D) Antennal lobe is demarcated with white dots and the neurons in the lineage by yellow dots. (B, C, E, F) Enlarged regions of cells from (B, C) larval and (E,F) adult brains labelled with antibodies against (B, E) dLim1 and (C,F) Cut. Scale bar = 10 μm. D = dorsal, L = lateral. [file 2042-1001-1-4-S5.TIFF]
